# Innovations in Intracoronary Imaging: Present Clinical Practices and Future Outlooks

**DOI:** 10.3390/jcm13144086

**Published:** 2024-07-12

**Authors:** Andreas Mitsis, Christos Eftychiou, Nikolaos P. E. Kadoglou, Konstantinos C. Theodoropoulos, Efstratios Karagiannidis, Athina Nasoufidou, Antonios Ziakas, Stergios Tzikas, George Kassimis

**Affiliations:** 1 Cardiology Department, Nicosia General Hospital, Nicosia 2029, Cyprus; chr6eft@gmail.com; 2 Medical School, University of Cyprus, Nicosia 2115, Cyprus; kadoglou.nikolaos@ucy.ac.cy; 3 First Department of Cardiology, AHEPA University Hospital, Aristotle University of Thessaloniki, 54636 Thessaloniki, Greece; ktheod2005@hotmail.com (K.C.T.); aziakas@auth.gr (A.Z.); 4 Second Department of Cardiology, Aristotle University of Thessaloniki, 54642 Thessaloniki, Greece; stratoskarag@gmail.com (E.K.); athinanassi@gmail.com (A.N.); gksup@yahoo.gr (G.K.); 5 Third Department of Cardiology, Aristotle University of Thessaloniki, 54636 Thessaloniki, Greece; tzikas@gmail.com

**Keywords:** intravascular imaging, intravascular ultrasound, optical coherence tomography, percutaneous coronary interventions, VH-IVUS, IB-IVUS, NIRS, OCT, PCI

## Abstract

Engaging intracoronary imaging (IC) techniques such as intravascular ultrasound or optical coherence tomography enables the precise description of vessel architecture. These imaging modalities have well-established roles in providing guidance and optimizing percutaneous coronary intervention (PCI) outcomes. Furthermore, IC is increasingly recognized for its diagnostic capabilities, as it has the unique capacity to reveal vessel wall characteristics that may not be apparent through angiography alone. This manuscript thoroughly reviews the contemporary landscape of IC in clinical practice. Focused on current methodologies, the review explores the utility and advancements in IC techniques. Emphasizing their role in clarifying coronary pathophysiology, guiding PCI, and optimizing patient outcomes, the manuscript critically evaluates the strengths and limitations of each modality. Additionally, the integration of IC into routine clinical workflows and its impact on decision-making processes are discussed. By synthesizing the latest evidence, this review provides valuable insights for clinicians, researchers, and healthcare professionals involved in the dynamic field of interventional cardiology.

## 1. Introduction

Intracoronary imaging (IC) has emerged as a valuable tool in contemporary interventional cardiology; providing detailed insights into coronary anatomy, plaque characteristics, and procedural guidance during percutaneous coronary intervention (PCI). With advancements in technology, several modalities such as intravascular ultrasound (IVUS), optical coherence tomography (OCT), and near-infrared spectroscopy (NIRS) have become integral components of cardiovascular interventions.

The pathogenesis of coronary artery disease (CAD) involves complex interactions between atherosclerotic plaque development, progression, and rupture [1]. Traditional diagnostic methods such as coronary angiography (CAG) provide valuable luminal information but offer limited insight into plaque composition and morphology. IC techniques, on the other hand, offer high-resolution, cross-sectional visualization of the vessel wall, enabling clinicians to assess plaque burden, identify vulnerable plaques, and guide optimal treatment strategies [2].

The primary modalities of interest—IVUS, OCT, and NIRS—each offer unique advantages and applications (Table 1). IVUS provides real-time, three-dimensional images of the vessel wall; aiding in the assessment of vessel size, plaque burden, and stent optimization. OCT, with its superior resolution, offers detailed visualization of plaque morphology; including fibrous cap thickness and lipid content, which is crucial for identifying high-risk plaques prone to rupture. NIRS complements these modalities by detecting lipid-rich plaques, contributing to the understanding of plaque vulnerability and guiding lipid-lowering strategies [3].

This review aims to explore the complex landscape of IC techniques; providing a detailed examination of its modalities, clinical applications, challenges, and future perspectives. By understanding the role of these imaging techniques in contemporary clinical practice, interventional cardiologists can optimize their use for improved patient outcomes in the management of CAD.

## 2. Intracoronary Imaging Modalities: Rationale, Advantages, and Limitations

### 2.1. Intravascular Ultrasound (IVUS)

IVUS provides high-resolution cross-sectional images of the coronary arteries; allowing detailed assessment of vessel dimensions, plaque morphology, and luminal narrowing. IVUS was introduced in the early 1990s [4], with subsequent technical advancements including the development of phased-array catheters [5,6]. This evolution prompted initial studies that compared IVUS image characteristics in peripheral and coronary vessels with histologic specimens and angiographic imaging. IVUS has been developed and improved significantly over the years and is now widely used in clinical practice for lesion assessment, stent optimization, and guiding PCI [7].

The IVUS catheter contains an ultrasound transducer that typically operates at frequencies ranging from 20 to 40 MHz. Conventional IVUS provides cross-sectional images of coronary arteries with an axial resolution typically ranging from 100 to 250 µm [8]. IVUS systems come in one of two types: mechanical or electronic. A mechanical IVUS uses a rotating transducer to generate images, while an electronic IVUS employs a stationary transducer with a rotating mirror for image acquisition. Both systems have their advantages and limitations, with mechanical systems offering higher resolution and better plaque delineation; but requiring rapid pullback and being more operator dependent. Electronic systems provide continuous image acquisition over longer distances; reducing operator variability, but at the cost of slightly lower resolution and higher expense [9]. Examples include Boston Scientific’s Atlantis SR Pro and iLab System for mechanical and electronic IVUS, respectively, as well as Volcano’s Eagle Eye Platinum IVUS and Philips’ OptiCross IVUS System.

**Figure 1:**
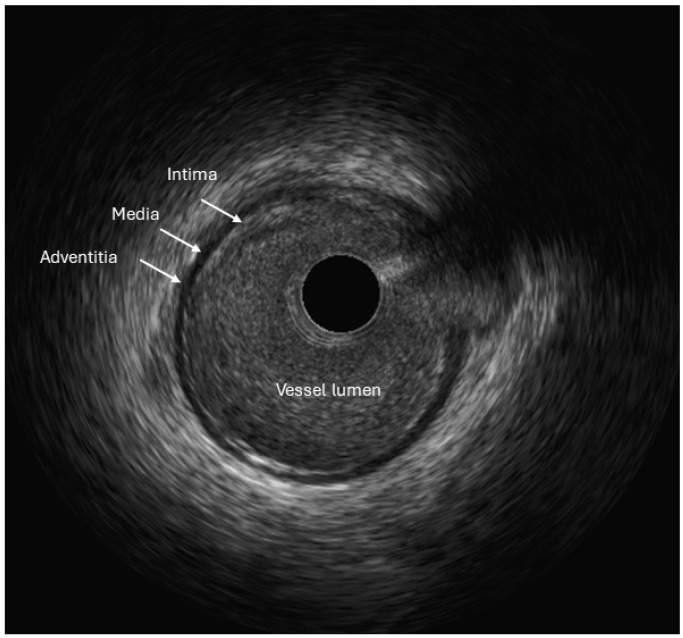
IVUS image showing the trilaminar appearance of a normal coronary arterial wall.

**Figure 2:**
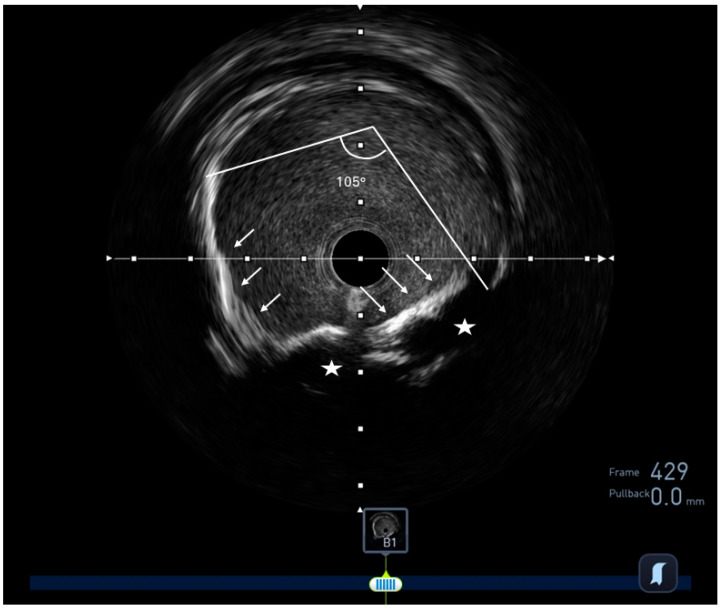
IVUS image of a calcified plaque with concentric calcium (white arrows) of 105 degrees. Note the acoustic shadow (white stars); a dark area behind calcific plaques.

A normal coronary artery on IVUS appears trilaminar; presenting intima as bright echo, media as dark, and adventitia again as bright (Figure 1). The atherosclerotic plaque, however, can be characterized based on the echo density into four categories. Soft (or necrotic) plaques are characterized by a lower echo signal than the outer membrane. These plaques have a large lipid pool, causing echo attenuation; and are considered vulnerable plaques, although they are prone to rupture. Calcific plaques have higher echo signals than the outer membrane and are considered stable plaques. Ultrasound waves cannot penetrate calcium, resulting in a dark area behind calcific plaques called “acoustic shadows” (Figure 2). Fibrous plaques (or hard noncalcified plaques) have moderate echo signals. These plaques are composed of packs of collagen fibres and sometimes can be misclassified as calcified regions. Finally, mixed (also called fibrofatty or fibrocalcific) plaques display two or more echo signals [9,10].

Furthermore, alongside the grayscale classifications derived from IVUS for atherosclerotic plaques, the following specific plaque phenotypes have been identified. The so-called attenuated plaques (AP) are identified by hypoechoic areas with deep echogenic attenuation, while echolucent plaques (ELP) are characterized by non-echoic or hypoechoic areas on IVUS, despite the absence of bright calcium. Both AP and ELP have been associated with a vulnerable plaque phenotype [11].

One of the key advantages of IVUS is its ability to measure the vessel size and plaque burden, aiding in the selection of appropriate stent size and length [12]. During PCI procedures, IVUS provides real-time guidance for optimal stent placement and expansion. It helps to assess the adequacy of stent apposition, identify calcification, detect edge dissections, and evaluate stent under-expansion or malapposition [13]. IVUS-guided PCI has been shown to improve clinical outcomes, including reduced rates of stent thrombosis and target vessel revascularization compared to angiography-guided PCI [14]. Additionally, IVUS can identify and treat bifurcation lesions more effectively by providing insights into the optimal stent technique (e.g., provisional stenting vs. two-stent strategy) [15]. 

Despite its advantages, IVUS has limitations; including the need for a blood-free vessel for optimal imaging and the inability to detect some types of plaques, such as microcalcifications or intraluminal thrombi. IVUS images also require interpretation skills, and there can be interobserver variability in plaque characterization [16]. Furthermore, IVUS is an invasive procedure with potential risks, such as arterial dissection or perforation.

### 2.2. Post-Processing Intravascular Ultrasound

Various algorithms have been developed to process IVUS images. Virtual histology (VH) is an IVUS-based post-processing modality that can create color-coded images of the coronary plaque and vessel lumen. While conventional grayscale IVUS has limited capacity to assess components within atherosclerotic lesions, VH-IVUS provides detailed analysis of plaque composition [17]. This technology uses radiofrequency signals obtained during IVUS imaging to characterize plaque components, such as fibrous tissue, necrotic core, calcification, and dense fibrosis. The resulting color-coded maps provide a “virtual” representation of the histological composition of the plaque, allowing clinicians to identify high-risk features such as lipid-rich plaques and thin-cap fibroatheromas. In VH-IVUS, the four basic tissue types constituting the plaque composition are: green for fibrous tissue, light green for fibrofatty tissue, red for necrotic tissue, and white for dense calcium [18]. The lesions, furthermore, can be classified as pathologic intimal thickening (PIT), fibrotic, fibrocalcific, thick-cap fibro atheroma (ThFA), or thin-cap fibro atheroma (TCFA). The VH-IVUS algorithm has been validated against histology from autopsy specimens and patients undergoing PCI, demonstrating high accuracy in detecting these components (autopsy: 79.7% fibrous, 81.2% fibrofatty, 85.5% necrotic core, 92.8% calcium; PCI patients: 87.1% fibrous, 87.1% fibrofatty, 88.3% necrotic core, 96.5% calcium) [19].

Integrated backscatter intravascular ultrasound (IB-IVUS) is another product of the evolution of post-processing techniques used by IVUS. It involves analyzing the backscattered ultrasound signals from tissues within the arterial wall. IB-IVUS provides information on the acoustic properties of tissues, allowing differentiation between different plaque components such as fibrous tissue, lipid content, and calcifications. IB-IVUS helps in identifying vulnerable plaques and guiding treatment strategies for patients with coronary artery disease [20]. In a recent validation study, IB-IVUS demonstrated an accuracy of 92% for fibrous, 91% for lipid-rich, and 95% for fibrocalcific components compared to histology [21].

Over the past decade, further advancements in IVUS technology have led to the development of radiofrequency (RF) signal-based IVUS systems for tissue characterization. The iMap-IVUS (Boston Scientific, Natick, MA, USA) stands out as a recent system utilizing a 40 MHz rotating single-element catheter. This system employs spectral RF analysis and a classification algorithm based on histological findings to categorize coronary plaque into fibrotic, lipidic, necrotic, or calcified components. Validation studies have demonstrated the accuracy of iMap-IVUS in tissue characterization, both in ex vivo and in vivo settings [22].

### 2.3. Optical Coherence Tomography (OCT)

OCT was first used in coronary interventions in the early 2000s [23]. Modern OCT systems utilize sophisticated near-infrared light sources and optical elements functioning at a wavelength of 1310 nm, with a low-coherence interferometer employed for measuring the backscattered light. Variations in tissue composition impact the reflection of light and the time taken for reflected energy to propagate; enabling the construction of a two-dimensional image depicting the optical scattering from the tissue structure [24]. OCT provides high-resolution, cross-sectional imaging with superior resolution compared to IVUS, enabling detailed visualization of coronary artery structures. This optical resolution of OCT is tenfold greater than that of IVUS, encompassing both longitudinal and temporal resolution [25]. 

**Figure 3:**
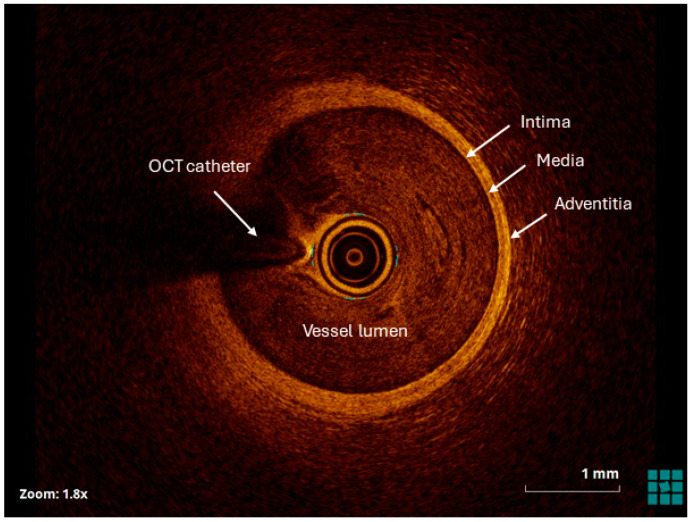
OCT image of a normal vessel. The normal vessel is characterized by three-layered appearance, including a high backscattering, thin intima, a low backscattering media and a high backscattering adventitia.

The increased resolution of the OCT in comparison with the IVUS provides detailed characterization of coronary plaques; including features such as fibrous cap thickness, lipid pools, and macrophage infiltration. This information is critical for identifying vulnerable plaques at risk of rupture and guiding treatment strategies [26]. OCT can differentiate between different types of plaque morphology, such as fibrous, fibrocalcific, and lipid-rich plaques, aiding in risk stratification and treatment selection. OCT interpretation requires experience and training [27]. The intima and adventitia contain plentiful collagen and elastin tissues, which exhibit bright appearances on OCT images owing to their inherent backscattering characteristics. Conversely, the medial layer contains a greater density of smooth muscle cells, which display limited reflective properties, resulting in a darker appearance on OCT images. This distinct “bright-dark-bright” pattern is indicative of the trilaminar structure typically observed in a healthy coronary artery (see Figure 3). However, the presence of atherosclerotic plaques disrupts this pattern, leading to alterations in tissue appearance [28].

**Figure 4:**
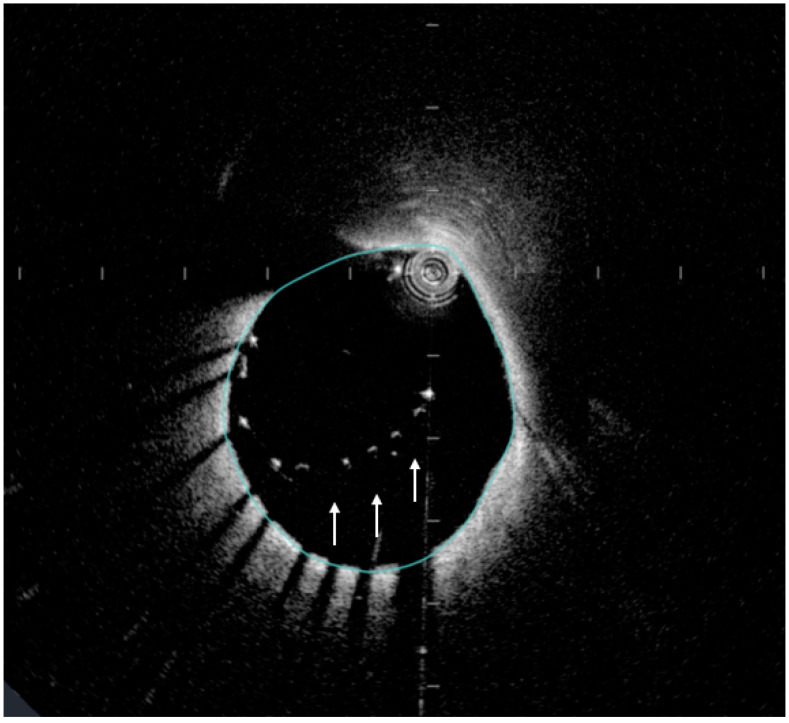
OCT image showing malapposition of the stent struts (white arrows) appearing as floating stent struts, with space between the struts and the plaque surface.

**Figure 5:**
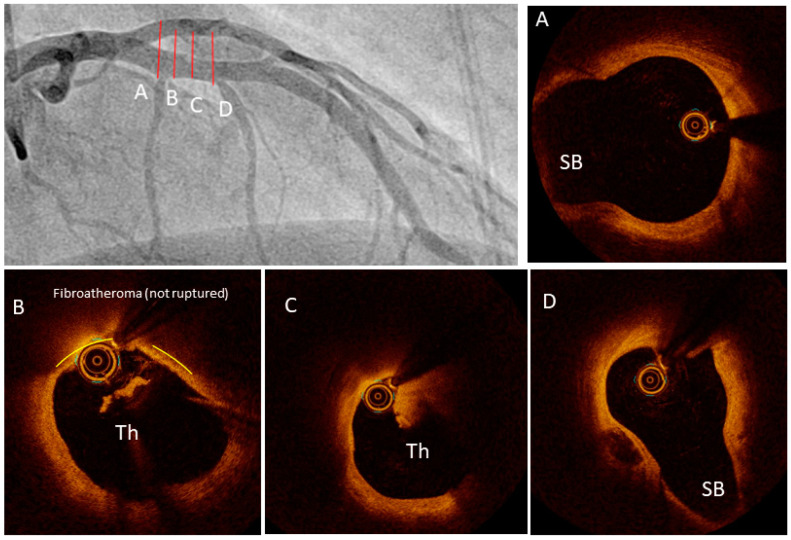
Angiography and OCT example of plaque erosion. The lines in the coronary angiography images (**A**–**D**) correspond to the respective cross-sectional OCT images. SB: side branch, Th: thrombus.

Furthermore, OCT is particularly useful for guiding coronary interventions assessing stent apposition, strut coverage, and identifying vulnerable plaques [29]. The high resolution of OCT allows for accurate measurement of luminal dimensions, stent area, and minimum lumen diameter, aiding in stent optimization and assessing stent integrity. During PCI, OCT provides real-time guidance for precise stent deployment and expansion. It allows for assessment of stent apposition against the vessel wall and detection of edge dissections or malapposition (Figure 4). OCT-guided PCI has been associated with improved clinical outcomes, including reduced rates of stent thrombosis and target lesion revascularization compared to angiography-guided PCI [30]. Additionally, OCT is valuable for evaluating complex lesions; for example, calcified lesions, where it can be used for precise identification of the extent of the calcium, the location of the calcium, the use of auxiliary devices and the post-stenting assessment of the result [31]. Finally, OCT can detect plaque erosion (Figure 5), which is a distinctly different underlying pathology of an acute coronary syndrome and therefore may merit tailored therapy [32].

Despite its superior resolution, OCT has limitations; including the need for blood removal or clearing agents for optimal imaging, which can affect procedural workflow. OCT is also limited by its shallow penetration depth compared to IVUS, making it less suitable for imaging in the presence of significant luminal thrombus or calcification. Interpretation of OCT images requires expertise, and there can be variability in image quality due to factors such as vessel motion or blood artifact.

### 2.4. Near-Infrared Spectroscopy (NIRS)

Near-infrared spectroscopy (NIRS) is a novel IC modality that provides information on lipid content within coronary plaques. The NIRS IVUS system includes a scanning near-infrared laser, a fiberoptic coronary catheter of similar size (3.2F monorail) and function to an IVUS catheter, along with an automated pullback and rotation device. A predictive algorithm estimates the probability that a lipid core plaque (LCP) is present at each interrogated region in the artery. NIRS measures the lipid core burden index (LCBI), which reflects the amount and distribution of lipid-rich plaques in the vessel [33]. The LCBI is provided as a quantitative summary metric of the presence of LCP in the entire scanned segment. NIRS is particularly useful for identifying lipid-rich plaques, which are associated with increased risk of plaque rupture and acute coronary syndrome (ACS). The color scale typically ranges from red to yellow to green to blue, representing different levels of lipid content within the plaque. Red indicates the highest lipid content, representing areas of high lipid core burden. Yellow indicates moderate lipid content. Green represents areas with low lipid content. Blue indicates minimal or no lipid content, often representing fibrotic or calcified plaques [34]. 

NIRS imaging can help in risk stratification and guiding treatment decisions, such as intensifying lipid-lowering therapy or considering invasive interventions [35]. During PCI, NIRS provides valuable information on the presence and distribution of lipid-rich plaques, aiding in lesion assessment and stent optimization [36]. It can identify areas of high lipid burden that may require specific treatment strategies, such as focal stenting or atherectomy. NIRS-guided PCI has the potential to improve outcomes by targeting lipid-rich plaques and reducing the risk of subsequent adverse events. Furthermore, NIRS provides quantitative information on the lipid content of coronary plaques, allowing for objective assessment of plaque vulnerability [37]. For this reason, the LCBI score indicates the extent of lipid deposition in the vessel, with higher scores associated with increased risk of plaque rupture [38]. Finally, NIRS imaging can complement other imaging modalities by providing additional information on plaque composition, particularly in cases where OCT or IVUS may not adequately capture lipid-rich plaques [34].

NIRS has limitations, including the need for a blood-free field for optimal imaging and limited depth of penetration, which may limit visualization of deeper plaques. Interpretation of NIRS data requires expertise, and there can be variability in measurements due to factors such as vessel curvature or motion artifacts. Additionally, NIRS imaging is still undergoing validation in clinical practice, and its role in routine clinical use is evolving.

## 3. Current Evidence

### 3.1. IVUS vs. Angiography

IVUS-guided PCI has emerged as superior to angiography-guided PCI. Several studies have compared the outcomes of IVUS-guided PCI versus angiography-guided PCI. Some of them did not demonstrate benefit [39,40,41] but most of them have shown a clear advantage of IVUS-guided PCI in both acute and mid-term clinical events [42,43,44,45,46] (Table 2). In the PROSPECT (Providing Regional Observations to Study Predictors of Events in the Coronary Tree) study, IVUS-guided PCI reduced primary endpoint events [Hazard Ratio (HR) 0.35, 95% confidence interval (CI) 0.18–0.67] and major adverse cardiovascular events (MACEs) (HR 0.53, 95% CI 0.37–0.76) at two years [42]; while the ADAPT-DES (Assessment of Dual Antiplatelet Therapy with Drug-Eluting Stents) trial, a randomized controlled trial (RCT) with 8583 patients, demonstrated that IVUS-guided PCI was associated with significantly lower rates of target lesion revascularization (TLR) at 1 year compared to angiography-guided PCI (4.5% vs. 5.7%, *p* = 0.02) [43]. The benefits of IVUS were especially evident in patients with ACS and complex lesions.

Additionally, in the IVUS-XPL (Impact of Intravascular Ultrasound Guidance on the Outcomes of Xience Prime Stents in Long Lesions) trial, when compared with angiography-guided stent implantation [44], IVUS-guided stent implantation resulted in a significantly lower rate of MACEs up to 5 years [45]. Finally, the ULTIMATE (Intravascular Ultrasound Guided Drug Eluting Stents Implantation in “All-Comers” Coronary Lesions) trial—an RCT with 1448 patients—demonstrated that IVUS-guided DES implantation significantly improved clinical outcome in all-comers [Target Vessel Failure (TVF) 2.9% vs. 5.4%, *p* = 0.0019], particularly for patients who had an IVUS-defined optimal procedure, compared with angiography guidance [46]. 

Many meta-analyses also confirmed the above finding. A study by Gao et al. included 4724 eligible patients from nine randomized trials. IVUS guidance was associated with a significantly lower risk of MACE [5.4% vs. 9.0%; relative risks (RR): 0.61, 95% CI 0.49–0.74, *p* < 0.001], cardiac death (0.6% vs. 1.2%; RR: 0.49, 95% CI 0.26–0.92, *p* = 0.03), target vessel revascularisation (TVR) (3.5% vs. 6.1%; RR: 0.58, 95% CI 0.42–0.80, *p* = 0.001), TLR (3.1% vs. 5.2%; RR: 0.59, 95% CI 0.44–0.80, *p* = 0.001), and definite/probable stent thrombosis (0.5% vs. 1.1%; RR: 0.45, 95% CI 0.23–0.87, *p* = 0.02) compared with angiography guidance [47]. Similarly, Ahn et al., conducted a meta-analysis with 26,503 patients from three randomized and 14 observational studies. IVUS-guided PCI was associated with a significantly lower risk of TLR (odds ratio [OR] 0.81, 95% CI 0.66 to 1.00, *p* = 0.046). Finally, the risk of death (OR 0.61, 95% CI 0.48 to 0.79, *p* < 0.001), MI (OR 0.57, 95% CI 0.44 to 0.75, *p* < 0.001), and stent thrombosis (OR 0.59, 95% CI 0.47 to 0.75, *p* < 0.001) were also reduced [48].

These results collectively suggest that IVUS-guided PCI is associated with improved clinical outcomes and a lower risk of adverse events compared to angiography-guided PCI.

### 3.2. OCT vs. Angiography

Several studies have investigated the outcomes of OCT-guided PCI compared to angiography-guided PCI (Table 3). The CLI-OPCI (Centro per la Lotta contro l’Infarto-Optimisation of Percutaneous Coronary Intervention) was a small observational study involving 145 patients, which demonstrated a significantly lower rate of MACEs at 1 year in the OCT-guided group compared to angiography-guided PCI (4.7% vs. 15.5%, *p* = 0.02) [49]. The authors showed that irregular findings by OCT imaging were common after ‘optimal’ PCI by angiographic standards. The largest, retrospective, CLI-OPCI II registry, showed a reduction in MACE at 2 years with OCT-guided PCI compared to angiography-guided PCI (5.5% vs. 15.7%, *p* = 0.01) [50].

The ILUMIEN (Observational Study of Optical Coherence Tomography [OCT] in Patients Undergoing Fractional Flow Reserve [FFR] and Percutaneous Coronary Intervention) trial—a non-randomized observational study with 418 patients with stable or unstable angina or non-ST-elevation myocardial infarction (NSTEMI)—found that OCT-guided PCI resulted in a significant reduction in TLR rates at 1 year compared to angiography-guided PCI (4.2% vs. 11.7%, *p* = 0.01) [54]. Physician decision-making was influenced by OCT findings, and it was more common in complex lesions. The decision made by physicians to respond to abnormalities detected by OCT following PCI identified a subgroup of coronary lesions exhibiting increased instances of malapposition, edge dissection, and stent under-expansion. 

The DOCTORS (Does Optical Coherence Tomography Optimize Results of Stenting) trial randomized 240 patients with NSTEMI to undergo OCT-guided PCI or fluoroscopy-guided PCI (angiography-guided group). The study showed a reduction in TVF at 1 year with OCT-guided PCI compared to angiography-guided PCI (3.7% vs. 8.3%, *p* = 0.02) [51]. 

The OCTOBER (Optical Coherence Tomography Optimized Bifurcation Event Reduction) trial studied patients with complex coronary-artery bifurcation lesions to undergo either OCT-guided PCI or angiography-guided PCI. OCT-guided PCI was associated with a lower incidence of MACE at 2 years than angiography-guided PCI (10.1% vs. 14.1%, (HR 0.70, 95% CI 0.50–0.98, *p* = 0.035) [52].

The large, recently published RCT ILUMIEN IV studied 2487 patients undergoing either OCT-guided PCI (n = 1233), or angiography-guided PCI (n = 1233). At 2-year follow-up, the minimum stent area (MSA) after PCI was 5.72 ± 2.04 mm^2^ in the OCT group and 5.36 ± 1.87 mm^2^ in the angiography group (mean difference, 0.36 mm^2^; 95% CI, 0.21 to 0.51; *p* < 0.001). Target vessel failure within 2 years was less common in the OCT group (7.4% vs. 8.2%, HR 0.90; 95% CI, 0.67 to 1.19; *p* = 0.45). Stent thrombosis within 2 years occurred in 6 patients (0.5%) in the OCT group and in 17 patients (1.4%) in the angiography group [53].

Moreover, a meta-analysis by Ali et al., including 6312 participants in 13 studies (eight randomized control trials and five observational studies) showed that OCT-guided PCI was associated with reduced all-cause [RR = 0.59, 95% CI (0.35, 0.97), *p* = 0.04] and cardiovascular mortality [RR = 0.41, 95% CI (0.21, 0.80), *p* = 0.009] compared to angiography-guided PCI [55]. 

These studies collectively suggest that OCT-guided PCI is associated with improved clinical outcomes—including lower rates of TLR, TVF, and MACE—compared to angiography-guided PCI.

### 3.3. OCT vs. IVUS vs. Angiography

Several large-scale registries, RCTs (Table 4), and meta-analyses have demonstrated OCT’s superiority to angiography and noninferiority to IVUS concerning both acute procedural results and mid-term clinical outcomes.

The OPUS-CLASS (OCT Compared with IVUS in a Coronary Lesion Assessment) study prospectively studied 100 patients, and showed that OCT provided accurate and reproducible quantitative measurements of coronary dimensions in the clinical setting [60]. Similarly, Bezerra et al. showed that OCT produces comparable reference lumen dimensions but demonstrates greater sensitivity in detecting disease severity, as well as improved identification of malapposition and tissue prolapse compared to IVUS [61].

The ILUMIEN II trial aims to determine whether OCT guidance results in a degree of stent expansion comparable to that with IVUS guidance [62]. This observational study compared the relative degree of stent expansion after OCT-guided stenting in the patients of the ILUMIEN study (n = 354) and IVUS-guided stenting in the patients of the ADAPT-DES study (n = 586). In the matched-pair analysis, the degree of stent expansion was not significantly different between OCT and IVUS guidance (median = 72.8% vs. 70.6%, respectively, *p* = 0.29). 

The largest, RCT ILUMIEN III (OPTIMIZE PCI) enrolled 450 patients, to undergo either OCT-guided, IVUS-guided or angiography-guided stent implantation [63]. At 1-year follow-up there were no significant differences in the rates of TLF (2.0% OCT, 3.7% IVUS, 1.4% angiography), MACE (9.8% OCT, 9.1% IVUS, 7.9% angiography), or any of the individual components of these outcomes among the groups [56].

The OPINION (OPtical frequency domain imaging vs. INtravascular ultrasound in percutaneous coronary InterventiON) study randomized 829 patients to either OCT-guided or IVUS guided PCI [57]. At 12-months OCT, guidance was non-inferior to IVUS guidance (HR 1.07, 95% CI 1.80; *p* non-inferiority = 0.042). The rate of TVF—a composite of cardiac death, MI, and ischemia-driven TVR at 12 months—was very low in patients undergoing OCT-guided PCI (5.2%) as well as in those undergoing IVUS-guided PCI (4.9%).

The iSIGHT (Optical Coherence Tomography Versus Intravascular Ultrasound and Angiography to Guide Percutaneous Coronary Interventions) aimed to examine whether PCI guided by OCT achieves stent expansion that is noninferior to that of IVUS guidance, and whether both imaging modalities result in superior stent expansion compared to an optimized strategy based solely on angiography [58]. The final MSA was not significantly different among the groups (OCT, 7.18 ± 2.66 mm^2^; IVUS, 6.97 ± 2.09 mm^2^; angiography, 7.26 ± 2.48 mm^2^, *p* = 0.820). The rates of long-term MACEs were low and not significantly different among the groups.

The most recent RENOVATE-COMPLEX-PCI (Randomized Controlled Trial of Intravascular Imaging Guidance versus Angiography-Guidance on Clinical Outcomes after Complex Percutaneous Coronary Intervention) study randomly assigned 1639 patients with complex coronary-artery lesions in a 2:1 ratio to undergo either intravascular imaging-guided PCI or angiography-guided PCI. In the intravascular imaging group, the choice between IVUS and OCT was the operators’ choice. During a median follow-up of 2.1 years, 76 patients (7.7%) in the intravascular imaging group and 60 patients (12.3%) in the angiography group experienced a primary endpoint event (HR 0.64; 95% CI, 0.45 to 0.89; *p* = 0.008). Cardiac-related deaths were recorded in 16 patients (cumulative incidence, 1.7%) in the intravascular imaging group and 17 patients (cumulative incidence, 3.8%) in the angiography group. Target-vessel-related MIs occurred in 38 patients (cumulative incidence, 3.7%) in the intravascular imaging group and 30 patients (cumulative incidence, 5.6%) in the angiography group. Clinically driven TVR was required for 32 patients (cumulative incidence, 3.4%) in the intravascular imaging group and 25 patients (cumulative incidence, 5.5%) in the angiography group [59].

## 4. Clinical Applications

### 4.1. Left Main Disease

Interventional cardiology faces considerable challenges when it comes to the PCI of the left main (LM) coronary artery. This scenario is particularly demanding due to the substantial amount of myocardium at risk and the technical intricacies associated with managing a complex bifurcation featuring sizable branches [64]. IC is considered, nowadays, a necessary tool in guiding LM treatment. In most cases, IVUS is favored over OCT due to its capability to conserve contrast dye and operate independently of blood clearance; a recognized constraint of OCT when assessing lesions near the ostium [65].

The Evaluation of XIENCE Versus Coronary Artery Bypass Surgery for Effectiveness of Left Main Revascularization (EXCEL) trial compared PCI with everolimus-eluting stents to coronary artery bypass grafting (CABG) for the treatment of left main coronary artery disease [66]. While not specifically focused on IVUS guidance, it provides important data on the safety and efficacy of PCI in the LM coronary artery. Findings from the EXCEL IVUS sub-study revealed a reduction in stent deformation, leading to lower occurrences of MACE among individuals who received IVUS guidance and optimization, with rates of 28% compared to 13.4%. Specifically, rates of LM-related MI were 18.9% versus 4.6%, and ischemia-driven TLR for LM was 19.6% versus 7.7% [67].

The Nordic-Baltic-British Left Main Revascularization Study (NOBLE) compared PCI with biolimus-eluting stents to CABG for the treatment of left main coronary artery disease [68]. Like EXCEL, while not focused on IVUS guidance, it provides relevant insights into the outcomes of PCI in the LM coronary artery. The IVUS sub-study of the NOBLE trial similarly highlighted the advantages of employing IVUS guidance, leading to a notable decrease in TLR (5.1% versus 11.6%, *p* = 0.01). However, intriguingly, this reduction did not correlate with a decrease in MACEs [69].

Several meta-analyses conducted during both the bare metal stent (BMS) and drug-eluting stent (DES) eras have demonstrated that IVUS guidance can reduce the risk of MACE in patients undergoing PCI for unprotected LM disease [70,71,72]. The ongoing OPTIMAL trial aims to provide definitive evidence about the clinical impact of IVUS-guidance during PCI to an unprotected LMCA [73]. The results of this study are expected early in 2026.

Based on IVUS studies, a minimum lumen area (MLA) exceeding 6.0 mm^2^ in Western populations demonstrates strong associations with a fractional flow reserve (FFR) above 0.80 [74]. The LITRO study—a prospective investigation—revealed that deferring LM lesions with an MLA surpassing 6.0 mm^2^ remained safe over a 2-year follow-up period, yielding outcomes comparable to those of patients who underwent revascularization. Additionally, individuals with an MLA below 6.0 mm^2^ who did not undergo revascularization experienced a notable rise in adverse events [75]. Given the existing data, lesions featuring an MLA smaller than 4.5 mm^2^ ought to be regarded as severe and warranting intervention. Conversely, deferring treatment and opting for conservative management for lesions with an MLA exceeding 6.0 mm^2^ is likely to be safe. However, lesions falling within the MLA range of 4.5 to 6.0 mm^2^ represent a “grey zone” and merit further evaluation with additional tests, such as intracoronary physiology assessments.

### 4.2. Bifurcation Lesions

Bifurcation coronary interventions present several challenges due to the complex anatomy and dynamic flow patterns at the bifurcation site. These challenges include difficulty in optimal stent positioning, inadequate stent expansion at the carina, risk of stent malapposition, and the potential for side branch compromise [76]. IC techniques—such as IVUS and OCT—offer valuable insights into lesion morphology, plaque distribution, and stent apposition; aiding in the assessment and optimization of bifurcation stenting.

Predicting TLR in bifurcation lesions poses a big challenge due to the multitude of complex bifurcation techniques and the presence and severity of side branch (SB) involvement, which complicate interpretations. Three mechanisms contributing to ISR were identified. Firstly, ISR resulted from stent under-expansion due to inadequate preparation of severely calcified or hard fibrous plaque lesions [77]. A plaque optimization technique (POT)—utilizing an appropriately sized balloon guided by intravascular imaging—could achieve more optimal and symmetrical stent strut dilation, thereby reducing ISR incidence [78]. Secondly, edge dissection following oversized stent deployment or aggressive post-dilatation increases restenosis risk [79]. IC guidance enables precise lesion assessment and device size selection, mitigating this complication. Thirdly, while bi-directional plaque extension into the ostial LM and proximal left anterior descending (LAD) artery is common in LM lesions observed via IVUS, mild to moderate lesions in the proximal LM may be overlooked for stent coverage, leading to proximal edge restenosis [80].

A recent meta-analysis with 7830 patients with bifurcation lesions showed that the incidence of MACEs with the IVUS-guided strategy were lower than those of patients with angiography-guided strategy at the early follow-up (OR = 0.55, 95% CI 0.42–0.70, *p* < 0.0001); while cardiac death, TVR, or TLR, and stent thrombosis were not statistically significant (OR = 0.68, 95% CI 0.34–1.35, *p* = 0.27; OR = 0.78, 95% CI 0.59–1.05, *p* = 0.10; OR = 0.36, 95% CI 0.12–1.04, *p* = 0.06). However, significant differences in cardiac death between IVUS-guided and angiographic-guided strategies were observed in the late follow-up (OR = 0.36, 95% CI 0.23–0.57, *p* < 0.00001) [81]. The ongoing RCT, IMPROVE (NCT04221815), aims to investigate the role of IVUS guided in complex PCI, including bifurcation lesions, and evaluate short-term and long-term clinical outcomes of the procedure [82].

The most recent joint consensus documents on bifurcation lesion PCI recommend the liberal use of OCT guidance [83]. OCT facilitates a meticulous assessment of bifurcation anatomy, plaque composition, and vulnerability; thereby influencing the revascularization strategy. Notably, specific parameters such as a bifurcation angle less than 50%, a length from the proximal branching point to the carina tip less than 1.70 mm, and the presence of lipid-rich plaque contralateral to the side-branch ostium area have been identified as independent predictors for side branch complications following bifurcation stenting [84]. The recently published OCTOBER trial including 1201 patients with complex coronary-artery bifurcation lesions, OCT-guided PCI was associated with a lower incidence of MACE at 2 years than angiography-guided PCI (10.1% vs. 14.1%, HR 0.70; 95% CI, 0.50 to 0.98; *p* = 0.035). Procedural-related complications occurred in 41 (6.8%) OCT-guided patients and 34 (5.7%) angiography-guided patients [52]. Of note, the use of OCT in bifurcation PCI has been recently proposed with the BOOM (Bifurcation and Ostial Optical coherence Mapping) technique [85]. This method promotes the accurate identification and mapping of the side-branch ostium using co-registration to reduce protrusion of stent struts into the main branch, while warranting full coverage of the ostium in the side branch.

### 4.3. In-Stent Restenosis (ISR)

**Figure 6:**
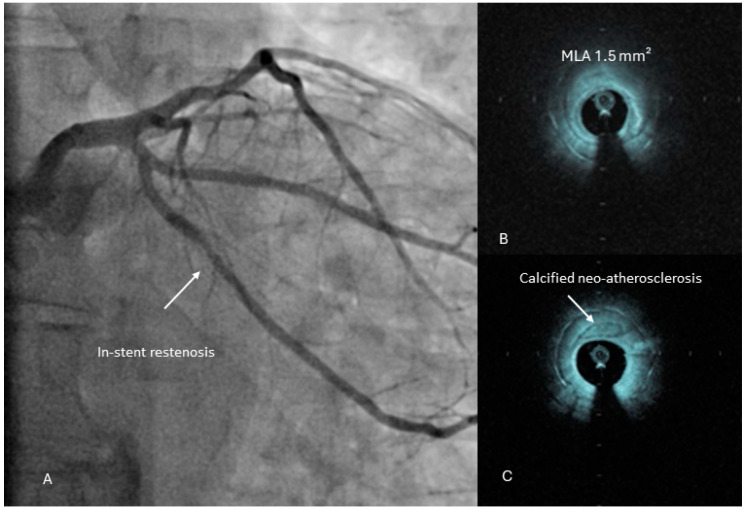
Angiography (**A**) and OCT (**B**,**C**) example of in-stent restenosis (ISR). The white arrow in (**A**) indicates the ISR. (**B**) shows the minimal lumen area (MLA) at the narrowest part of the previously implanted stent. In (**C**), the white arrow indicates the calcified neoatherosclerosis as the cause of ISR.

Even with the advent of DES aimed at addressing neointimal hyperplasia following BMS implantation, a notable proportion of patients continue to experience ISR; especially as PCI tackles progressively more complex lesions. ISR is classified as luminal narrowing of >50% in a stented coronary segment or within 5 mm of a stent edge [86]. IC offers distinct perspectives on different types of stent-related mechanical problems (stent under-expansion, under-sizing, stent fracture, stent edge dissection, nonoverlapping stents) that contribute to ISR [87]. Practically, IC can assist in refining the stenting procedure to prevent such issues. Furthermore, when restenosis arises, IC can guide management by addressing the underlying biological promoter mechanisms (neointimal hyperplasia, neoatherosclerosis) (Figure 6) [88]. Ultimately, it can also be employed to assess the outcomes of reintervention procedures.

IVUS-guided PCI is known to reduce ISR for both BMS [70,89,90] and DES [43,91,92,93]. IVUS provides detailed information about stent geometry, including stent apposition, malapposition, and under-expansion. Assessment of stent expansion is critical for identifying inadequate stent deployment, which may contribute to ISR. But even if ISR occurs, IVUS can guide the need for reintervention and evaluate the efficacy of treatment strategies, with the accurate measurement of luminal dimensions; including MLA and diameter stenosis, which are essential for determining the severity of ISR and guiding treatment decisions [94]. IVUS can help identify the location and extent of restenosis, determine the appropriate balloon size, balloon type (scoring balloon, cutting balloon), and inflation pressure for angioplasty; and assess the results of balloon angioplasty or auxiliary procedures such as intravascular lithotripsy [95].

Similarly, OCT plays a crucial role in the prevention and management of ISR. OCT can easily detect the mechanical contributing factors of ISR and optimize the PCI procedure [96]. Furthermore, it can differentiate the underlying biological promoter mechanism—i.e., neointimal hyperplasia from neoatherosclerosis—as a cause of ISR and guide the proper management [97,98,99]. Neointimal hyperplasia refers to the excessive growth of smooth muscle cells within the stent, which can lead to luminal narrowing [100]. OCT provides high-resolution imaging that enables precise assessment of the composition and morphology of the neointima. Neointimal hyperplasia typically appears as homogeneous tissue with relatively low backscatter intensity on OCT images. On the other hand, neoatherosclerosis involves the formation of lipid-rich plaques within the stent, such as de novo atherosclerosis. These plaques may lead to in-stent restenosis due to luminal obstruction and thrombosis [101]. OCT can differentiate neoatherosclerotic plaques from neointimal hyperplasia based on their distinct morphological features. Neoatherosclerotic plaques often exhibit lipid-laden regions with signal-rich, low-intensity areas on OCT images, as well as the presence of cholesterol crystals, macrophage infiltration, and thin fibrous caps [102].

### 4.4. Stent Thrombosis (ST)

IC is crucial in the diagnosis and treatment of stent thrombosis (ST); offering high-resolution, detailed insights into the mechanisms underlying this complication [103]. Both techniques, IVUS and OCT, provide precise visualization of the stent and surrounding vessel, enabling clinicians to identify specific causes of ST. They can detect stent malapposition and stent under-expansion, as both conditions can create areas of turbulent blood flow, predisposing to thrombus formation [104]. With its better resolution, OCT can furthermore identify issues such as edge dissections, where the vessel lining is torn at the stent edges, creating sites for thrombus initiation. It also excels in detecting neointimal hyperplasia, an excessive growth of tissue within the stent, which can narrow the stent lumen and contribute to ST. Additionally, OCT can reveal delayed endothelialization, where the stent struts remain exposed due to insufficient endothelial cell coverage, increasing the risk of clot formation [105].

By identifying these specific issues, IC informs targeted treatment strategies. For instance, detecting stent malapposition or under-expansion may prompt post-dilation with a balloon to ensure proper stent deployment. Recognizing edge dissections might lead to the placement of additional stents to cover the dissected area. Understanding the extent of neointimal hyperplasia can guide decisions on adjunctive therapies, such as drug-eluting stents or systemic antithrombotic medications [106].

Overall, IC enhances the understanding of ST etiology, guides precise interventional strategies, and aids in monitoring the effectiveness of these interventions; ultimately improving patient outcomes by reducing the incidence and recurrence of ST.

### 4.5. Spontaneous Coronary Artery Dissection (SCAD)

**Figure 7:**
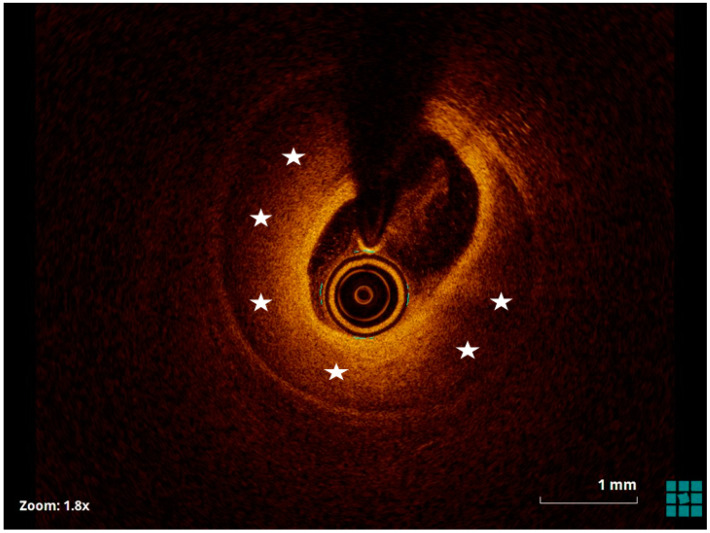
OCT image of spontaneous coronary dissection (SCAD) showing the intramural hematoma (white stars).

IC is essential in the diagnosis, management, and follow-up of spontaneous coronary artery dissection (SCAD) [107]. Both IVUS and OCT can help to identify the dissection flap, differentiate true and false lumens, and assess the presence and extent of intramural hematoma (Figure 7) [108]. OCT with its higher resolution allows for an even more detailed visualization of the vessel layers and can detect subtle features of SCAD that might be missed by IVUS. OCT is particularly useful in identifying the entry point of the dissection and the precise length and depth of the dissection plane. It can also aid in distinguishing between SCAD and other coronary pathologies, such as atherosclerotic plaque rupture [109].

By providing these detailed images, IC helps clinicians to make informed decisions regarding the management of SCAD. It assists in determining whether conservative management is sufficient or if PCI is necessary. Furthermore, during PCI, IC can guide stent placement to ensure optimal coverage of the dissection and minimize the risk of complications. In follow-up, IC can monitor the healing process, assess stent apposition and expansion, and detect any recurrence of dissection or other complications [110]. Overall, IVUS and OCT are indispensable tools in the comprehensive management of SCAD; enhancing diagnostic accuracy, guiding therapeutic strategies, and improving patient outcomes. However, the use of IC can potentially worsen a dissection or cause a new intimal tear. Therefore, the use of OCT in evaluating SCAD should be carefully considered by weighing its diagnostic advantages against the procedural risks [111].

### 4.6. Chronic Total Occlusion (CTO)

In chronic total occlusions (CTO), IVUS plays a crucial role in guiding PCI by providing detailed anatomical information about the occluded vessel; including vessel size, plaque burden, calcification, and vessel course [112]. IVUS helps in assessing the feasibility of CTO PCI, guiding wire and device selection, optimizing lesion preparation, and evaluating stent deployment. Additionally, IVUS can identify potential complications such as perforations, dissections, and stent under-expansion during the procedure [113]. Finally, IVUS can streamline re-entry techniques, offer immediate confirmation of the positioning of a second wire within the distal true lumen, and pinpoint the optimal re-entry point, thereby circumventing calcified segments and areas with a significant gap between the false and true lumen [114].

Several trials have investigated the role of IVUS in CTO PCI. The AIR-CTO study aims to compare angiographic endpoints at one-year follow-up after a DES implantation guided by IVUS or angiography in patients with CTO lesions. The IVUS-guided stenting of the CTO lesion was associated with less late lumen loss (LLL 0.28 ± 0.48 mm vs. 0.46 ± 0.68 mm, *p* = 0.025) and a lower incidence of “in-true-lumen” stent restenosis (3.9% vs. 13.7%, *p* = 0.021) [115]. In the CTO-IVUS RCT, a total of 402 patients with CTOs were randomly allocated to either the IVUS-guided group (n = 201) or the angiography-guided group (n = 201). Following a 12-month follow-up period, the incidence of cardiac death did not show a significant difference between the IVUS-guided group (0%) and the angiography-guided group (1.0%; *p* by log-rank test = 0.16). However, the rates of MACEs were notably lower in the IVUS-guided group compared to the angiography-guided group (2.6% versus 7.1%; *p* = 0.035; HR, 0.35; 95% CI, 0.13–0.97) [116]. Interestingly, in the recent RENOVATE-COMPLEX-PCI trial, among the 319 patients with complex CAD characterized by chronic total occlusions (CTOs). The trial’s subgroup analysis demonstrated a notable reduction in the primary outcome with the use of intravascular imaging (including both IVUS and OCT) compared to angiography guidance (HR: 0.30; 95% CI: 0.13–0.71) [59].

## 5. Contemporary Clinical Guidelines

All the above IC techniques play a crucial role in guiding PCI and optimizing outcomes in patients with CAD. Guidelines from major cardiovascular societies emphasize the importance of IC in various clinical scenarios to enhance procedural success and long-term efficacy. The American College of Cardiology (ACC) and American Heart Association (AHA) guidelines recommend IC in select cases. IVUS has a class IIa recommendation in PCI guidance, especially in unprotected LM disease and complex coronary stenting to reduce ischemic events. OCT in PCI guidance is promoted as an alternative of IVUS with the same IIa recommendation, except in ostial left main disease. Finally, both IVUS and OCT have a IIa recommendation in cases of stent failure to investigate the mechanism of stent failure [117].

Similarly, the European Society of Cardiology (ESC) advocates for the use of IC in guiding unprotected LM PCI or when to optimize stent implantation (Class IIa) [118,119]. More recently, IC received class IIa recommendation to guide all PCI interventions. IC, and preferably OCT, is also recommended in lesions with ambiguous angiographic features (Class IIb recommendation) [120]. Of note—despite there being a broad consensus among guidelines regarding the utility of IC in optimizing PCI outcomes—differences may exist in specific recommendations based on regional practices, available technologies, and expert consensus. 

Current guidelines support the use of intracoronary physiology such as fractional flow reserve (FFR) and/or instantaneous wave-free ratio (iFR) as the gold standard approach to proceed with coronary revascularization in patients with intermediate coronary stenosis [117,118,119,120]. The specific added value of intracoronary imaging in this context lies in its ability to provide detailed morphological information about the coronary artery plaque. IC techniques such as IVUS and OCT can characterize plaque composition; and identify features such as thin cap fibroatheromas, calcification, and plaque burden, which are not discernible with FFR alone. This detailed imaging aids in the assessment of lesion severity, guides optimal stent placement, and ensures proper stent expansion and apposition; thereby improving procedural outcomes and reducing the risk of restenosis and stent thrombosis. Additionally, IC can help to identify vulnerable plaques that might benefit from preventive strategies, even if the FFR is not indicative of ischemia. 

The added value of non-invasive methods such as coronary flow reserve (CFR), as attained by enhanced transthoracic Doppler echocardiography (E-Doppler TTE) and Positron Emission Tomography (PET), in assessing the hemodynamic severity of coronary artery disease lies in their ability to provide comprehensive and integrative information about both epicardial and microvascular function. CFR (the maximal capability to increase flow) along with blood-flow mapping of the related epicardial vessel by E-Doppler TTE can discriminate between segmental stenosis, diffuse athero- and pure microcirculatory coronary dysfunction, thus capturing the overall functional state of the coronary arteries and microvasculature with no radiation exposure; as recently demonstrated [121]. PET imaging offers precise quantification of myocardial blood flow and perfusion, allowing for the identification of regional and global perfusion defects and microvascular dysfunction. These non-invasive methods reduce procedural risks, improve patient comfort, and provide a more detailed physiological assessment, enhancing diagnostic accuracy and guiding more tailored therapeutic strategies [122,123,124].

## 6. Challenges and Limitations

Despite its numerous benefits, IC is not without challenges. Technical considerations, interpretation of images, and the need for specialized training are factors that clinicians must navigate [125]. The adoption and proficiency in IC techniques require a substantial learning curve due to the complexity and precision needed for accurate interpretation. Initially, practitioners must acquire a theoretical understanding of the technology, imaging principles, and indications for use. Hands-on training with supervised procedures is essential, where new users perform imaging under the guidance of experienced operators. Simulation-based training can also be beneficial, providing a risk-free environment to practice image acquisition and interpretation [126]. Structured mentorship programs are crucial, involving step-by-step guidance from experienced colleagues. Regular feedback and case discussions help to refine skills and build confidence. Additionally, workshops and dedicated courses offered by professional societies provide intensive training opportunities. Online resources—including webinars, instructional videos, and virtual simulators—offer flexible learning options to complement practical experience. Assessment of proficiency should be ongoing, incorporating both technical skills in image acquisition and interpretative accuracy. Performance metrics and benchmarks can be used to evaluate progress. Proficiency in intracoronary imaging is typically achieved after performing a significant number of supervised procedures, with continuous learning and experience enhancing the operator’s competence and confidence over time [127].

Additionally, the cost-effectiveness and widespread adoption of these technologies remain areas of discussion. The first significant challenge is the interpretation of imaging data, as it requires specialized training and expertise to accurately assess plaque characteristics and lesion severity. Image artifacts, such as shadowing or attenuation, can also distort the image quality, potentially leading to misinterpretation [128]. Another challenge is the invasiveness of the procedure and the increased procedural complexity associated with IC techniques as it involves threading a catheter through the coronary arteries, which can pose risks such as dissection or perforation. Furthermore, IC increases procedural time, and it can potentially impact patient throughput in a busy catheter laboratory. 

Furthermore, the applicability of IC techniques in ACS faces several challenges. Firstly, the urgent nature of ACS necessitates rapid decision-making, which may limit the time available for detailed imaging. Additionally, the hemodynamic instability of patients can complicate the procedure, increasing the risk of adverse events during imaging. Finally, the need for specialized equipment and expertise also restricts the widespread use of these techniques, particularly in resource-limited settings [129]. 

Additionally, the cost associated with IC modalities and the need for dedicated equipment may limit widespread adoption [130]. Standardization of imaging protocols and interpretation criteria across centers is also crucial for ensuring consistency and reliability of results [131]. Moreover, IC imaging is typically performed concurrently with angiography, exposing patients and staff to additional radiation. Lastly, the evolving landscape of IC technologies presents a challenge in keeping up with the latest advancements and determining which modality best suits the clinical scenario [132].

## 7. Future Perspectives

The landscape of IC is continuously evolving, driven by emerging technologies and innovative approaches. As we look towards the future, several exciting developments and potential advancements are on the horizon. Novel IC modalities are being developed, such as intravascular photoacoustic imaging [133] and intracoronary near-infrared fluorescence imaging [134]. These technologies offer enhanced visualization of coronary anatomy and plaque characteristics, potentially revolutionizing our understanding of CAD. Additionally, the integration of three-dimensional (3D) imaging into clinical practice holds promise for improved spatial visualization and accurate assessment of vessel geometry. Real-time 3D reconstruction during procedures could enhance procedural guidance and stent optimization [135]. Of note, combined imaging and physiology techniques—such as FFR and intravascular thermography—provide comprehensive assessments of both anatomical and functional parameters. Integration of these modalities, in the concept of functional imaging, could offer a more holistic approach to CAD diagnosis and treatment [136].

Bioresorbable scaffolds (BRS) present a significant advancement in coronary interventions, offering potential benefits such as restoration of vessel pulsatility and reduced risk of late stent thrombosis [137]. With the emergence of BRS for coronary interventions, there is a need for imaging techniques to assess scaffold apposition, expansion, and degradation over time. IVUS and OCT are likely to play a crucial role in optimizing BRS implantation and monitoring long-term outcomes. Moreover, as BRS undergo gradual resorption, longitudinal imaging with IVUS and OCT becomes essential for tracking scaffold degradation and assessing vessel healing over time [138]. This continuous monitoring can help clinicians identify any adverse events, such as scaffold discontinuities or late acquired malapposition, and guide appropriate management strategies [139].

Artificial intelligence (AI) and machine learning (ML) algorithms have the potential to transform IC analysis [140,141]. These technologies can aid in rapid and automated plaque characterization, vessel measurements, and identification of high-risk features. AI-driven predictive models may enable personalized risk stratification based on imaging data, patient characteristics, and clinical outcomes. This could lead to tailored treatment strategies, optimizing patient care and outcomes. Furthermore, AI-powered systems could assist interventionalists in real-time decision-making during procedures. Automated lesion recognition and guidance for optimal stent sizing and positioning could improve procedural success rates [142].

Finaly, IC may guide targeted therapies aimed at specific plaque phenotypes. Tailored treatment strategies based on plaque composition could lead to more effective interventions and improved outcomes. Longitudinal imaging studies tracking changes in plaque morphology over time could provide insights into plaque progression and vulnerability [143]. Understanding dynamic changes in plaques may guide preventive strategies. Combining intracoronary imaging with circulating biomarkers could enhance risk prediction and prognosis. Biomarker-guided imaging protocols may offer a comprehensive approach to CAD management [144].

In conclusion, the future of IC holds tremendous promise with the integration of emerging technologies, AI-driven analysis, and targeted therapies. These advancements are poised to revolutionize CAD diagnosis, treatment guidance, and risk prediction. Continued research and collaboration will drive the translation of these innovations into clinical practice, ultimately benefiting patient care and outcomes.

## 8. Conclusions

IC represents a pivotal advancement in modern interventional cardiology, providing unparalleled insights into CAD pathophysiology and guiding personalized treatment strategies. Throughout this manuscript, we have focused on the diverse applications of IC, from its role in diagnosing CAD and optimizing PCI to assessing plaque characteristics and predicting outcomes. Our exploration of current clinical practices and emerging technologies highlights the evolving landscape of intracoronary imaging. Moving forward, it is evident that integrating advanced imaging modalities, AI, and targeted therapies will be instrumental in enhancing patient care. As IC continues to evolve, we emphasize the importance of ongoing research and collaboration to harness its full potential. In conclusion, IC stands as a cornerstone in modern cardiology; offering a wealth of opportunities for improved diagnosis, treatment, and prognosis in patients with CAD.

## Figures and Tables

**Table 1:** A comparison of the major characteristics of intravascular ultrasound (IVUS), optical coherence tomography (OCT), and near-infrared spectroscopy (NIRS).

Characteristics	IVUS	OCT	NIRS
Imaging Principle	Ultrasound	Near-infrared light	Near-infrared light
Resolution (μm)	100–250	10–20	5–10
Tissue Penetration Depth (mm)	4–8	1–3	2–4
Sheath compatibility	5F or larger	6F or larger	5F or larger
Visualizes	Vessel lumen, media, plaque	Detailed vessel structures	Lipid-rich plaques
Image Quality	Good	Excellent	Good
Calcium Visualization	Less effective	Limited due to signal attenuation	Less effective
Guidance in Stent Deployment	Helpful	Very helpful	Limited
Plaque Characterization	Limited	Detailed	Lipid Core Identification
Assessment of Stent Apposition	Good	Excellent	Limited
Vulnerable Plaque Detection	Limited	Possible	Yes
Procedure Time	Longer	Shorter	Moderate
Cost	Moderate	Higher	Lower

**Table 2:** Randomized controlled trials investigating intravascular ultrasound (IVUS)-guided PCI versus invasive coronary angiography (ICA)-guided PCI.

Study	Year	Design	Patients (n)	Follow-Up	Primary Endpoint	Secondary Endpoints	Main Findings
HOME DES IVUS [39]	2010	RCT (IVUS-guided PCI vs. ICA)	210	18 months	Death, MI	TLR, TVR	IVUS-guided PCI did not significantly reduce death (HR 1.15, 95% CI 0.59–2.22) or MI (HR 1.18, 95% CI 0.55–2.54) compared to angiography.
PROSPECT [42]	2011	Prospective Cohort (IVUS-guided PCI)	697	2 years	Death, MI, cardiac arrest	MACE	IVUS-guided PCI reduced primary endpoint events (HR 0.35, 95% CI 0.18–0.67) and MACE (HR 0.53, 95% CI 0.37–0.76) at two years.
Kim et al. [40]	2013	RCT (IVUS-guided PCI vs. ICA)	430	1 year	MACE	TLR, stent thrombosis	IVUS-guided PCI non-inferior for MACE (HR 0.82, 95% CI 0.51–1.30) and stent thrombosis (HR 0.82, 95% CI 0.35–1.91) compared to angiography.
AVIO [41]	2013	RCT (IVUS-guided PCI vs. ICA)	284	2 years	MACE	TLR, stent thrombosis	No differences were observed in cumulative MACE (16.9% vs. 23.2%), cardiac death (0% vs. 1.4%), MI (7.0% vs. 8.5%), TLR (9.2% vs. 11.9%) or TVR (9.8% vs. 15.5%), respectively in the IVUS vs. angio-guided groups. A benefit of IVUS optimized DES implantation was observed in complex lesions in the post-procedure minimal lumen diameter.
ADAPT-DES [43]	2014	RCT (IVUS-guided PCI vs. ICA)	8583	2 years	TVF	Stent thrombosis, MI	IVUS-guided PCI reduced TVF (HR 0.72, 95% CI 0.55–0.94) and stent thrombosis (HR 0.47, 95% CI 0.25–0.87) compared to angiography. The benefits of IVUS were especially evident in patients with ACS and complex lesions.
IVUS-XPL [44]	2019	RCT (IVUS-guided PCI vs. ICA)	1400	5 years	TLF	MACE	IVUS-guided PCI reduced TLF (HR 0.58, 95% CI 0.37–0.92) and MACE (HR 0.63, 95% CI 0.45–0.88) compared to angiography.
ULTIMATE [46]	2019	RCT (IVUS-guided PCI vs. ICA)	1448	1 year	MACE	TLF, stent thrombosis	IVUS-guided PCI reduced TVFs (HR 0.53, 95% CI 0.31- 0.901; *p* = 0.019) compared to angiography.

ACS: acute coronary syndrome; CI: confidence interval; HR: hazard ratio; ICA: invasive coronary angiography; IVUS: intravascular ultrasound; MACE: major adverse cardiac events; MI: myocardial infarction; PCI: percutaneous coronary intervention; RCT: randomized controlled trial; TLR: target lesion revascularization; TVR: target vessel revascularization; TLF: target lesion failure; TVF: target vessel failure.

**Table 3:** Randomized controlled trials comparing optical coherence tomography (OCT)-guided PCI versus invasive coronary angiography (ICA)-guided PCI.

Study	Year	Design	Patients (n)	Follow-Up	Primary Endpoints	Secondary Endpoints	Main Findings
DOCTORS [51]	2021	RCT (OCT vs. ICA)	240	2 years	Post PCI fractional flow reserve	Procedural complications and type 4a periprocedural MI	OCT-guided PCI resulted in a significantly higher fractional flow reserve post procedure (0.94 ± 0.04 vs. 0.92 ± 0.05, *p* = 0.005). There was no significant difference in the rate of type 4a myocardial infarction (33% in the OCT-group vs. 40% in the angiography-guided group, *p* = 0.28).
OCTOBER [52]	2023	RCT (OCT vs. ICA)	1201	2 years	MACE	Cardiac death, TLR, MI	OCT-guided PCI resulted to lower rates of MACE (HR 0.70, 95% CI 0.50–0.98, *p* = 0.035).
ILUMIEN IV [53]	2023	RCT (OCT vs. ICA)	2487	2 years	MSA, TVF	Cardiac death, TLR, MI	OCT-guided PCI showed lower rates of TVF (7.4% vs. 8.2%, HR 0.90; 95% CI, 0.67–1.19; *p* = 0.45).

CI: confidence interval; HR: hazard ratio; ICA: invasive coronary angiography; MACE: major adverse cardiac events; MI: myocardial infarction; PCI: percutaneous coronary intervention; RCT: randomized controlled trial; TLR: target lesion revascularization; TVF: target vessel failure.

**Table 4:** Randomized controlled trials comparing optical coherence tomography (OCT)-guided PCI versus intravascular ultrasound (IVUS)-guided PCI versus invasive coronary angiography (ICA)-guided PCI.

Study	Year	Design	Patients (n)	Follow-Up	Primary Endpoints	Secondary Endpoints	Main Findings
ILUMIEN III [56]	2016	RCT (OCT vs. IVUS vs. ICA)	450	1 year	Post PCI MSA	MACE	No significant difference in MACE (*p* = 0.68, 95% CI 0.78–1.25), TLR (*p* = 0.54, 95% CI 0.87–1.42), TVR (*p* = 0.71, 95% CI 0.82–1.29), and device success (*p* = 0.92, 95% CI 0.91–1.13) between OCT-guided and IVUS-guided PCI. OCT was superior to ICA and non-inferior to IVUS regarding post-PCI MSA.
OPINION [57]	2018	RCT (OCT vs. IVUS)	829	1 year	TVF	MACE, cardiac death, MI, TLR, TVR	TVF occurred in 21 (5.2%) of 401 patients undergoing OCT-guided PCI, and 19 (4.9%) of 390 patients undergoing IVUS-guided PCI, demonstrating non-inferiority of OCT-guided PCI to IVUS-guided PCI (HR 1.07, 95% CI 1.80; *p* non-inferiority = 0.042).
iSIGHT [58]	2021	RCT (OCT vs. IVUS vs. ICA)	156	2 years	Post PCI MSA	MACE, cardiac death, MI, TLR, TVR	The final MSA was not significantly different among the groups (OCT, 7.18 ± 2.66 mm^2^; IVUS, 6.97 ± 2.09 mm^2^; ICA, 7.26 ± 2.48 mm^2^, *p* = 0.820).
RENOVATE-COMPLEX-PCI [59]	2023	RCT (OCT or IVUS vs. ICA)	1639	2 years	TVF	MACE, cardiac death, MI, TLR, TVR	Intravascular imaging–guided PCI resulted to a lower risk of a composite of death from cardiac causes (1.7 vs. 3.8%), target-vessel–related MI (3.7 vs. 5.6%), or clinically driven TVR (3.4 vs. 5.5%) than angiography-guided PCI.

CI: confidence interval; HR: hazard ratio; ICA: invasive coronary angiography; IVUS: intravascular ultrasound; MACE: major adverse cardiac events; MI: myocardial infarction; RCT, randomized controlled trial; TLR: target lesion revascularization; TVR: target vessel revascularization; TVF: target vessel failure.

## Data Availability

Not applicable.
